# Atrial size and function post orthotopic heart transplantation – CMR and ECHO study

**DOI:** 10.1186/1532-429X-11-S1-P272

**Published:** 2009-01-28

**Authors:** Nishant Kalra, Vincent L Sorrell

**Affiliations:** Sarver Heart Center, University of Arizona, Tucson, AZ USA

**Keywords:** Leave Atrial, Right Atrial, Atrial Contraction, Atrial Size, Orthotopic Heart Transplantation

## Background

The conventional orthotopic heart transplantation (OHT) (biatrial) results in atrial enlargement. The variability of atrial size and function in these patients has not been previously reported.

## Objectives

1. Determine the variability in the post-OHT size and function of left and right atria; 2. Analyze the relationship of donor and native atria; 3. Compare cardiac MRI (CMR) with transthoracic echocardiogram (TTE).

## Methods

This is a retrospective observational study. Nine patients who had undergone OHT via biatrial anastomosis technique and had both CMR as well TTE within one month of each other were selected. Using the optimized apical 4-chamber views in both TTE and CMR cine images (SSFP), endocardial borders of the native and donor, right atrial (RA) and left atrial (LA) free wall and septum were traced from base to the valvular annulus and areas were determined in late systole when atrial volumes are maximal and late diastole when atrial volumes are minimal. RA and LA function was assessed quantitatively using percentage of change in cavity area or fractional area change (FAC). FAC was defined using the formula ([end-systolic area] - [end-diastolic area])/[end-systolic area] × 100). Native and donor areas were also combined to calculate the total area of the right and left atria. Comparison was made between the TTE and CMR values. Data for continuous variables are expressed as mean value +/- SD.

## Results

See Tables 1 and 2. TTE and CMR were performed within 6 + 9 days of each other. The native atria (retained atrial cuff) are usually larger than the donor atria on the left and almost equal on right. The total LA area is usually > total RA area. 7 patients have LA total area > 24 cm2 and 4 of these patients also have RA total area > 24 cm2. TTE showed a good correlation with CMR, however within individual patients, there were both over- and under-estimation of atrial areas and TTE consistently underestimated native atrial areas. Native atrial function was consistently worse than donor atrial function. Overall total LA function was better than total RA function. TTE consistently overestimated the FAC compared to CMR in all except the native right atrial group.

**Table 1:** Atrial Area (mean +/- SD) measured by CMR and TTE

	CMR Systole	CMR Diastole	TTE Systole	TTE Diastole
Total Left Atrial Area	30.6 +/- 13.3	27.7 +/- 13.8	30.5 +/- 12.6	22.2 +/- 11.1
Total Right Atrial Area	24.9 +/- 8.1	23.6 +/- 7.7	20.6 +/- 7.7	18.5 +/- 7.5
Donor Left Atrial Area	11.9 +/- 3.7	8.5 +/- 3.9	13.1 +/- 3.1	8.3 +/- 2.8
Donor Right Atrial Area	12.4 +/- 3.1	11.3 +/- 3.0	11.8 +/- 3.4	9.5 +/- 2.8
Native Left Atrial Area	18.7 +/- 10.1	19.2 +/- 10.4	17.4 +/- 9.8	13.9 +/- 8.3
Native Right Atrial Area	12.5 +/- 5.8	12.3 +/- 5.4	8.8 +/- 5.1	8.9+/4.8

**Table 2:** Fractional Area Change (mean +/- SD) measured by CMR and TTE

	CMR	TTE
Total Left FAC	10.3 +/- 8.9	28.8+/12.2
Total Right FAC	4.9 +/- 8.9	10.6 +/- 7.8
Donor Left FAC	29.7 +/- 12.8	36.6 +/- 13.4
Donor Right FAC	8.4 +/- 13.1	17.6 +/- 16.4
Native Left FAC	-3.9 +/- 13.0	20.9 +/- 18.0
Native Right FAC	0.6 +/- 10.1	-8.2 +/- 25.1

## Discussion

**Figure 1:**
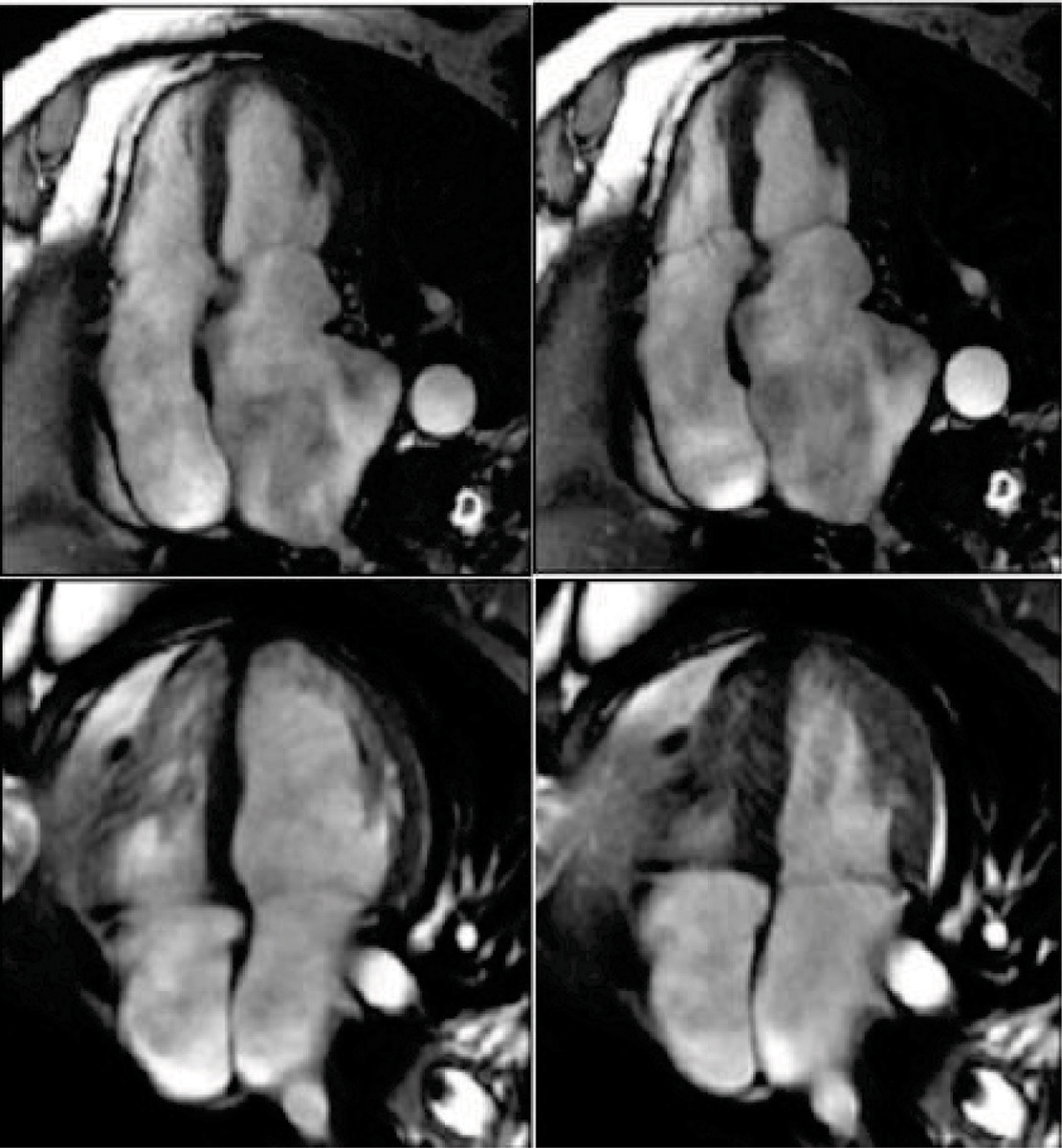
Figure 1

Left atrial volumes consistently predict outcome in most patient groups studied. LA size is frequently severely distorted after OHT and is dependent upon extent of retained pulmonary vein cuffs or associated donor lung preparation, individual cardiac surgeon preferences, and cardiac recipient organ status. Knowing the variations in atrial sizes may provide a new avenue for clinical investigation (Figure 1). A CMR atrial area <24 cm2 is considered normal, but rarely present in this post-OHT patient subgroup.

It appears that the retained native atrium does not contribute to atrial contraction. This resulted in a negative total FAC% in 45% (4/9) of our patients. Given the large retained native atria, and its poor contribution to atrial contraction, this report suggests that minimizing this atrial reservoir may be valuable and warrants additional study. The underestimation of native atrial size reported by TTE is most likely due to apical fore-shortening of the true long-axis and the far field ultrasound imaging.

## Conclusion

This observational study suggests that despite a generally reasonable correlation between TTE and CMR measures of OHT atria, significant differences exist. Furthermore, because of suspected intra-atrial (donor versus native) mechanical dysynchrony, some patients surprisingly had a negative FAC% suggesting that the native atria is larger during donor atrial contraction – acting as a non-functioning reservoir. The association of this to the clinical outcome has yet to be determined.

